# An Atypical Presentation of Sporadic Jejunal Burkitt's Lymphoma

**DOI:** 10.1155/2016/3605813

**Published:** 2016-09-08

**Authors:** Pratik Naik, James Wang, Michael J. Brazeau, Domingo Rosario

**Affiliations:** ^1^Department of Internal Medicine, William Beaumont Army Medical Center (WBAMC), 5005 North Piedras Street, El Paso, TX 79920, USA; ^2^Division of Gastroenterology, Department of Internal Medicine, William Beaumont Army Medical Center (WBAMC), 5005 North Piedras Street, El Paso, TX 79920, USA; ^3^Department of Pathology, William Beaumont Army Medical Center (WBAMC), 5005 North Piedras Street, El Paso, TX 79920, USA

## Abstract

Burkitt's lymphoma is a very aggressive type of B-cell NHL with replication approaching 100%. Primary gastrointestinal lymphoma is rare. In our case, a 24-year-old male initially presented with symptomatic anemia. He was initially evaluated with colonoscopy and EGD, both of which were unremarkable. A capsule endoscopy was then performed to further evaluate his significant anemia which revealed friable inflamed ulcerated mass in the jejunum. A push enteroscopy was then performed to obtain tissue from the jejunal mass. Biopsy results and immunohistochemical stains were consistent with Burkitt's lymphoma. PET/CT scan revealed only jejunal involvement. Treatment consisted of bowel resection prior to chemotherapy due to concern for perforation with chemotherapy. Patient achieved complete remission after the treatment.

## 1. Introduction

Primary gastrointestinal (GI) non-Hodgkin's lymphoma (NHL) is rare, and it is the most common extranodal lymphoma site. NHL most occurs in the stomach followed by ileum and ileocecum [1]. Diffuse B-cell Lymphoma is the most common type of GI lymphoma occurring in all of GI tract [1]. Only 2% of sporadic GI malignancies occur in the small intestine [2]. Primary sporadic jejunal Burkitt's lymphoma (BL) is extremely rare. There are no specific symptoms of GI NHL and diagnosis remains difficult. Initial presentation with obstruction and perforation has been documented in the past in all types of NHL [3]. Lymphomas can present as multiple ulcerated lesions mimicking inflammatory bowel disease and most specifically Crohn's disease if the small bowel is primarily involved [4]. Our case is unique in that the initial presentation was misleading due to iron deficiency anemia with questionable ulcerated mass in jejunum, which was suggestive of Crohn's disease.

## 2. Case Report

**Figure 1:**
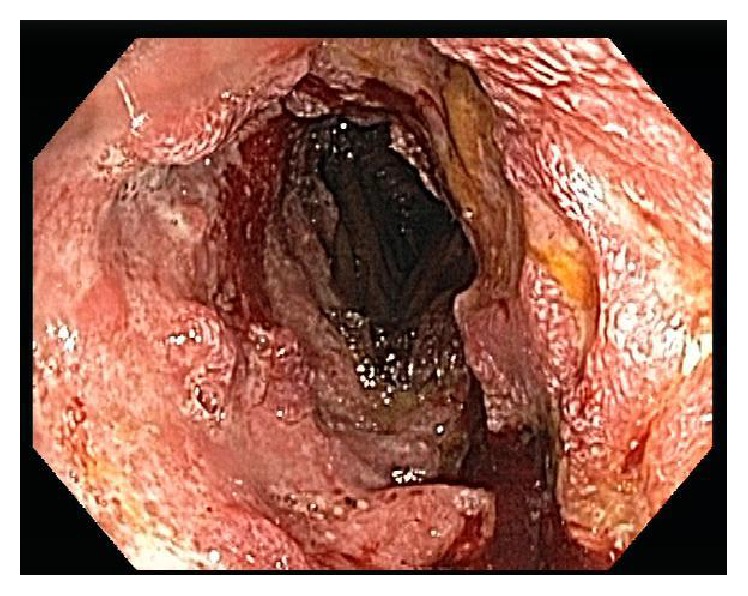
A push enteroscopy shows a 10 cm long, friable, ulcerated mass in the 3rd part of the jejunum.

**Figure 2:**
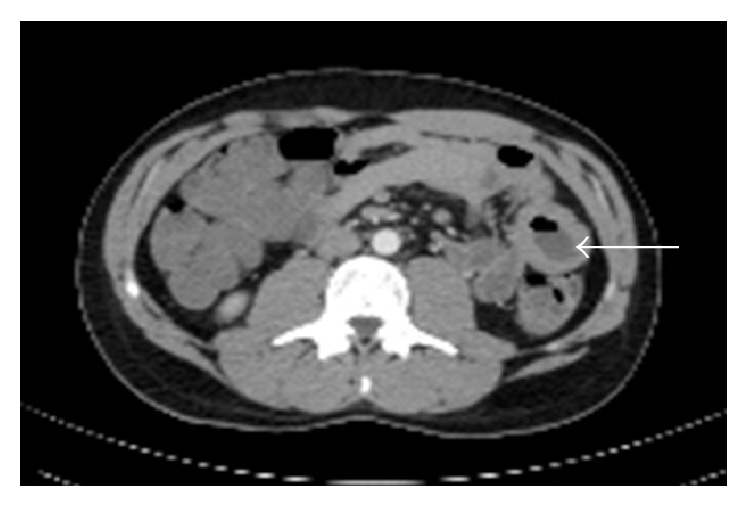
A computed tomography (CT) of abdomen shows lumen thickening with narrowing in distal jejunum (arrow).

**Figure 3:**
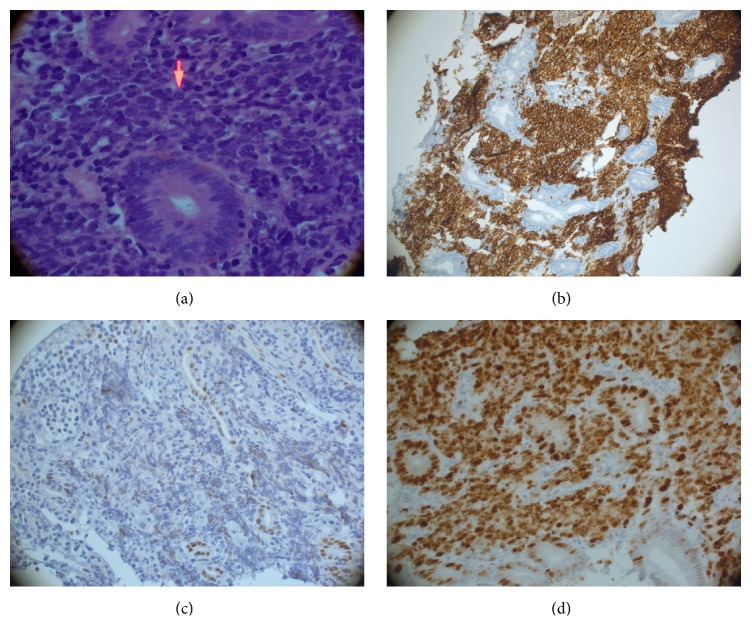
(a) High power (400x), H&E (hematoxylin and eosin), medium to large lymphoid cells invading the lamina propria featuring multiple nucleoli (arrow) consistent with high grade lymphoma (Burkitt's lymphoma shows multiple nucleoli). (b) CD20 highlights lamina propria atypical lymphoid infiltrate, consistent with high grade B-cell lymphoma. (c) Negative staining for MYC immunohistochemical stain. (d) 100% nuclear positivity for Ki-67, consistent with high proliferation rate.

**Figure 4:**
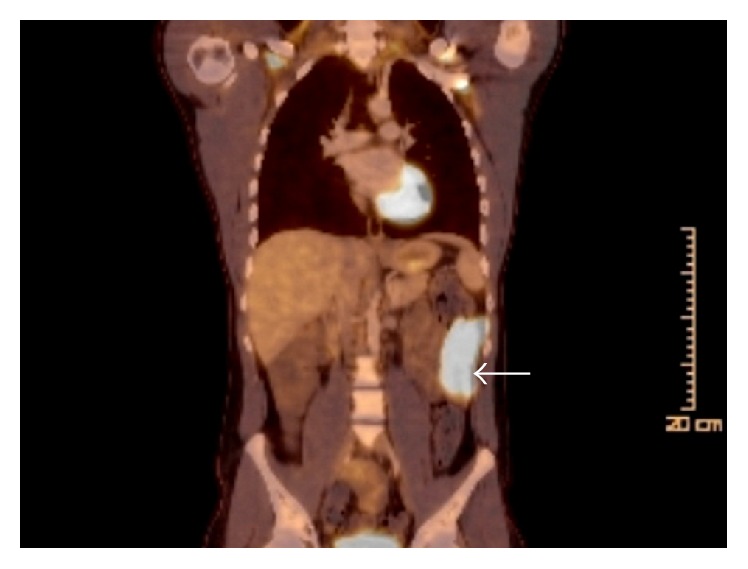
PET scan shows FDG avid small bowel segment with circumferential thickening (arrow) with no evidence of distant disease.

A 24-year-old US Army soldier was found to be anemic on routine blood work while he was in Kuwait. He was referred to gastroenterology for further evaluation. A colonoscopy and an esophagogastroduodenoscopy (EGD) performed in Kuwait were unremarkable. One month later, he started experiencing weakness, dyspnea, reduced exercise tolerance, dizziness, and lightheadedness. At that time, he was not experiencing any diarrhea, abdominal pain, night sweats, melena, or hematochezia. After further evaluation, the patient was found to be severely anemic with hemoglobin of 5.6 g/dL, MCV of 77 fL, iron saturation < 2%, and ferritin of 2.0 ng/mL. He received 4 units of PRBCs in Kuwait. The patient was immediately medically evacuated to Fort Bliss, TX, for further evaluation and treatment of his anemia. A colonoscopy and EGD were repeated with normal findings. Due to concern for obscure GI bleed, a capsule endoscopy was performed, which showed a 10 cm long questionable jejunal lesion. A push enteroscopy was performed and a biopsy was taken from a friable, ulcerated mass in the 3rd part of the jejunum (Figure 1). A computed tomography (CT) of abdomen was also obtained which showed lumen thickening with narrowing in distal jejunal area (Figure 2). At this point, his presentation was suggestive of Crohn's disease. The initial pathology report was notable for a malignant process and was positive for CD20 (Figure 3(b)) and CD 79a immunohistochemical stains. Further immunostaining revealed a Ki-67 proliferative fraction approaching 100%, reflecting a very rapidly growing neoplasm (Figure 3(d)). Other staining types were notable for CD10 and BCL6 positivity and negative for MUM1, BCL2, CD44, CD5, Cyclin D1, c-Myc (Figure 3(c)), and TdT. He was also screened for anti-HIV antibody and anti-EBV IgM and IgG, which were seronegative. His other evaluations consisted of a positron emission tomography (PET) scan with avid uptake localized to the jejunum only (Figure 4) and a bone marrow biopsy without evidence of lymphoma involvement. He was given a diagnosis of BL, Stage 1EB (WHO classification). Treatment consisted of prophylactic bowel resection followed by adjuvant chemotherapy (CODOX-M/IVAC). Patient achieved complete remission after the treatment.

## 3. Conclusion/Discussion

Burkitt's lymphoma is a very aggressive type of B-cell NHL with replication approaching 100%. BL has three clinical forms: endemic, sporadic, and immunodeficiency-associated. The sporadic variant is seen in the US and Western Europe. Sporadic type BL comprises 30% of pediatric lymphomas and less than 1% of adult non-Hodgkin lymphomas in the US [5]. The GI tract is the predominant site of extranodal lymphoma involvement. Primary gastrointestinal lymphoma is rare usually secondary to the widespread nodal diseases constituting only about 1%–4% of all gastrointestinal malignancies [1]. These lymphomas have been described in ileum, stomach, cecum and/or mesentery, kidney, testis, ovary, breast, bone marrow, and CNS. BL is mostly seen in pediatric population, and it is associated with Epstein-Barr virus (EBV) and HIV/AIDS patients [1].

The presentation and symptoms of lymphomas of the small intestine are nonspecific and can be misleading. BL can present as GI bleeding, bowel obstruction, intussusceptions, colicky abdominal pain, nausea, vomiting, and weight loss. GI lymphomas can mimic acute appendicitis and Crohn's disease [5]. Primary BL in the jejunum has been reported in literature with initial presentation of obstruction and perforation [3]. Primary intestine lymphomas with multiple ulcers can have similar presentation to Crohn's disease. Classically Crohn's disease involves the terminal ileum and ileocecal valve with multiple small punctiform, rounded nodules, or superficial erosions known as aphthoid lesions. Over a period of time, the erosions become confluent and give rise to larger longitudinal ulcers, known as serpiginous ulcers [6]. Bowel wall thickening with luminal narrowing can be seen on CT abdomen in Crohn's disease [7], as was also seen in our patient. There is an increased risk of lymphomas with chronic inflammatory disease such as rheumatoid arthritis, Hashimoto's thyroiditis, and Sjogren's syndromes [8]. Prior large population cohort studies have demonstrated no increased risk of B-cell lymphomas in inflammatory bowel disease and risk of NHL is similar to general population [9, 10].

Tissue biopsies are critical for diagnosis. Criteria for diagnosis BL include immunohistochemical stains CD 20 (+), CD10 (+), BCL-6 (+), and BCL-2 (−) and a proliferative fraction >95% as per WHO classification [11]. c-Myc gene translocation can be present; however, 10–15% BL can be c-Myc negative [12]. In our case, the patient had all the criteria for BL as per WHO classification but was negative for c-Myc loci, which makes our case more unique. Double hit lymphomas or B-cell lymphomas, unclassifiable with intermediate features between diffuse large B-cell lymphoma (DLBCL) and BL, usually will show variably strong positivity for BCL-2 and larger more pleomorphic nuclei instead of medium-sized nuclei. Both of these features (i.e., pleomorphic nuclei and BCL2 positivity) were absent in the current case, making the diagnosis of BL more likely. Also, DLBCL usually shows a Ki-67 proliferation rate of about 65–70% (the current case is essentially 100%). In addition, CD44 is most likely to be expressed in CD10+ positive DLBCL and it was negative in the current case, further supporting the diagnosis of BL [13].

Radiological findings of small intestinal lymphoma are nonspecific and biopsy is required for definitive diagnosis. BL usually presents as a bulky mass in the right lower quadrant on a CT scan [1]. Evaluation and diagnosis of small bowel lymphomas have been more effective with the use of capsule endoscopy and double-balloon technique of push-and-pull enteroscopy. Initial evaluation such as biopsies and other interventions can be performed using a double-balloon enteroscopy which can potentially limit need for surgeries [1]. In our case, we were able to identify the jejunal lesion on a capsule endoscopy. Staging is usually done by a PET scan and/or a CT scan of chest, abdomen, and pelvis. Bone marrow or CNS involvement portends poor prognosis.

BL is a rapidly growing tumor and prophylactic surgery can be considered if a high risk of perforation or other complications are suspected. The role of elective surgery in the management of intestinal lymphoma is not well defined. In a retrospective study, 9% (92 of 1062) of the patients with primary GI lymphomas developed perforation, of which 55% occurred after chemotherapy. The risk of perforation was higher with aggressive B-cell lymphomas such as DLBCL and BL compared to indolent B-cell lymphomas. DLBCL was the most common lymphoma (59%) and small intestine was the most common site (59%) associated with perforation [14]. A recent systemic review noted overall survival benefit with surgery and no reported increased postoperative morbidity or mortality [15]. Other prior studies also reported better outcomes and event-free survival with surgical resection followed by chemotherapy [16, 17]. Prospective studies are needed to further define benefits of resection before chemotherapy but it is a reasonable approach for localized intestinal lymphomas. However, there have been no reports of cure with resection alone and aggressive early chemotherapy is required even after a surgical resection.

Short term multiregimen chemotherapy such as CALGB 10002, CODOX-M/IVAC, EPOCH, and HyperCVAD with rituximab is highly effective with a complete remission rate up to 90% [2, 18]. In a small study involving 19 HIV-free patients, the use of a less toxic dose adjusted EPOCH (etoposide, prednisone, vincristine, cyclophosphamide, and adriamycin) plus rituximab (DA-REPOCH) led to an event-free survival of 96% and an overall survival of 100% [19].
